# Supplementary material for: Effect of Plateau pika on Soil Microbial Assembly Process and Co-Occurrence Patterns in the Alpine Meadow Ecosystem
Source: Microorganisms. 2024 May 26;12(6):1075. doi: 10.3390/microorganisms12061075 (PMC11205797; doi:10.3390/microorganisms12061075)
Supplement: Supplementary file 1 [file microorganisms-12-01075-s001.zip › Supplementary file.pdf]

Supplementary Information for

Effect of *Plateau pika* on Soil Microbial Assembly Process and Co-Occurrence Patterns in the Alpine Meadow Ecosystem

Xiangtao Wang [1,2,*,†], Zhencheng Ye [3,†], Chao Zhang [3,4] and Xuehong Wei [2,*]

[1] School of Life Sciences, Guizhou Normal University, Guiyang 550025, China
[2] Qiangtang Alpine Grassland Ecosystem Research Station, Tibet Agricultural and Animal Husbandry University, Nyingchi 860000, China
[3] State Key Laboratory of Soil Erosion and Dryland Farming on the Loess Plateau, Northwest A&F University, Xianyang 712100, China; yezhencheng1996@nwafu.edu.cn (Z.Y.); zhangchaolynn@163.com (C.Z.)
[4] Institute of Soil and Water Conservation, Chinese Academy of Sciences and Ministry of Water Resources, Xianyang 712100, China
* Correspondence: wangxt@xza.edu.cn (X.W.); weixuehong@21cn.com (X.W.)
† These authors contributed equally to this work.

This PDF file includes:

Tables S1-S3

Figures S1-S13

Table S1 The detailed sampling information.

Patch	Area/m^2	Number of quadrats	Longitude	Latitude
S1	4	3	29° 55' 11"E	92° 21' 41"N
S2	56	5	29° 55' 33"E	92° 22' 05"N
S3	100	6	29° 54' 48"E	92° 22' 24"N
S4	210	10	——	——
S5	400	12	29° 54' 50"E	92° 22' 12"N
S6	592	15	29° 54' 48"E	92° 22' 24"N

Table S2 Alpha and beta diversity of bacterial and fungal communities on different patches. Each mean in table has an associated SE. The lowercase letters indicate significant differences between the different patches at the 0.05 level of the least significant difference (LSD) test. Chao1: Chao1index; Richness: the richness of ASV; Shannon: Shannon–Wiener index. S1-S6 represents patches ranging in size from small to large.

	Treatment	Chao1	Richness	Shannon	Beta diversity
**Bacteria**	S1	4207±864 bc	3682±743 bc	7.32±0.20 a	0.52±0.03
	S2	6803±818 a	5449±483 a	7.41±0.16 a	0.67±0.04
	S3	5318±477 ab	4652±367 ab	7.43±0.07 a	0.68±0.03
	S4	4808±334 b	4115±261 b	7.27±0.04 a	0.70±0.02
	S5	5630±294 ab	4796±195 ab	7.41±0.03 a	0.60±0.02
	S6	3348±277 c	2885±252 c	6.65±0.11 b	0.65±0.01
**Bacterial generalist**	S1	996±156 a	920±157 a	6.26±0.18 a	0.46±0.04
	S2	1153±86 a	1004±100 a	6.29±0.13 a	0.54±0.03
	S3	969±79 a	876±81 a	6.14±0.12 a	0.54±0.02
	S4	989±79 a	874±82 a	6.13±0.12 a	0.58±0.02
	S5	1152±49 a	1039±53 a	6.36±0.07 a	0.48±0.01
	S6	699±66 b	613±64 b	5.72±0.15 b	0.60±0.01
**Bacterial specialist**	S1	725±139 b	580±78 c	5.14±0.00 a	0.56±0.04
	S2	1582±350 a	1174±226 a	5.30±0.25 a	0.72±0.03
	S3	1213±177 ab	1045±162 ab	5.51±0.27 a	0.71±0.02
	S4	976±99 b	808±86 bc	5.22±0.17 a	0.73±0.02
	S5	1057±108. b	843±91 abc	5.08±0.16 a	0.64±0.01
	S6	846±67 b	733±56 c	5.00±0.18 a	0.64±0.01
**Fungi**	S1	291±29 c	184±9 c	3.42±0.09 ab	0.72±0.03
	S2	369±51 abc	266±51 ab	3.55±0.13 ab	0.76±0.03
	S3	327±38 bc	206±18 bc	3.65±0.18 ab	0.79±0.02
	S4	444±30 a	279±26 a	3.36±0.21 b	0.72±0.02

	S5	305±20 c	183±11 c	3.35±0.09 b	0.71±0.01
	S6	377±26 ab	204±10 c	3.77±0.11 a	0.84±0.01
**Fungal generalist**	S1	15.9±2.1 b	11.0±0.5 d	0.99±0.41 ab	0.98±0.00
	S2	22.2±4.2 ab	17.8±3.1 abc	0.82±0.11 b	0.70±0.08
	S3	35.8±11.2 a	20.6±1.9 ab	1.16±0.14 ab	0.84±0.05
	S4	31.5±3.1 a	21.5±1.4 a	1.37±0.14 a	0.63±0.03
	S5	20.4±1.5 b	16.1±1.2 bcd	1.13±0.12 ab	0.68±0.03
	S6	19.9±1.7 b	15.4±1.0 cd	1.47±0.12 a	0.86±0.02
**Fungal specialist**	S1	98.3±19.8 b	60.3±7.2 ab	2.55±0.09 abc	0.89±0.03
	S2	111±16 ab	80.4±15.7 a	2.64±0.24 abc	0.93±0.02
	S3	97.2±6.7 b	67.1±5.7 ab	2.89±0.2 ab	0.92±0.02
	S4	143±19 ab	73.1±5.8 ab	2.2±0.22 c	0.93±0.01
	S5	102±11 b	56.4±3.2 b	2.53±0.13 bc	0.91±0.01
	S6	152±18 a	83.8±7.3 a	3.13±0.11 a	0.86±0.01

Table S3 The relationship between different soil properties and patch area. TN: total nitrogen; TP: total phosphorus; AP: available phosphorus; SOC: soil organic carbon; AK: available potassium; NO_3^-: nitrate nitrogen; NH_4^+: ammonium nitrogen; Heterogeneity: the habitat heterogeneity, measured as measured as Euclidean distances of these soil properties among samples.

Independent Variable	Dependent Variable	Slope	R^2	*p*
Disturbed area	TN	-0.3193	0.1711	< 0.01
	TP	-0.00704	0.01148	0.4542
	SOC	-9.698	0.2457	< 0.001
	AK	-9.699	0.1148	< 0.05
	AP	-0.33335	0.2342	< 0.001
	NO_3^-	-3.81	0.07976	< 0.05
	NH_4^+	-10.156	0.1137	< 0.05
	pH	-0.04668	0.02343	0.2835
	Moisture	-3.735	0.2742	< 0.001
	Heterogeneity	-6.46	0.3366	< 0.001

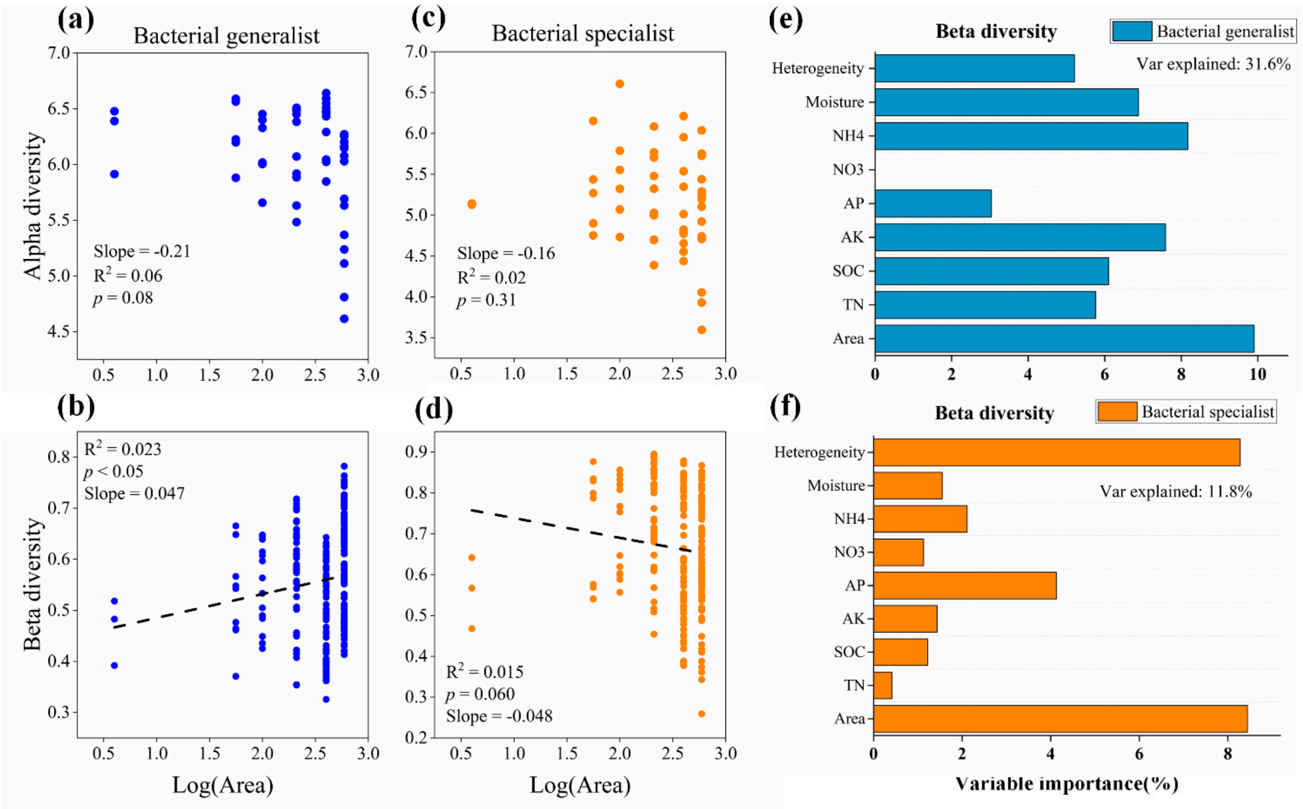

Figure S1 The effects of patch area on the alpha and beta diversity of bacterial generalist and specialist, and their important predictors. Panels a and b are for generalist alpha and beta diversity, respectively; panels c and d are for specialist alpha and beta diversity, respectively. Alpha diversity was measured as the Shannon-Weiner index. Beta diversity was measured as average pairwise Bray–Curtis dissimilarities among samples. Panels e and f are for the fraction of the variation in beta diversity of soil bacterial generalist and specialist explained by environmental and spatial predictors. TN: total nitrogen; TP: total phosphorus; AP: available phosphorus; SOC: soil organic carbon; AK: available potassium; NO_3^-: nitrate nitrogen; NH_4^+: ammonium nitrogen; Heterogeneity: the habitat heterogeneity, measured as measured as Euclidean distances of these soil properties among samples.

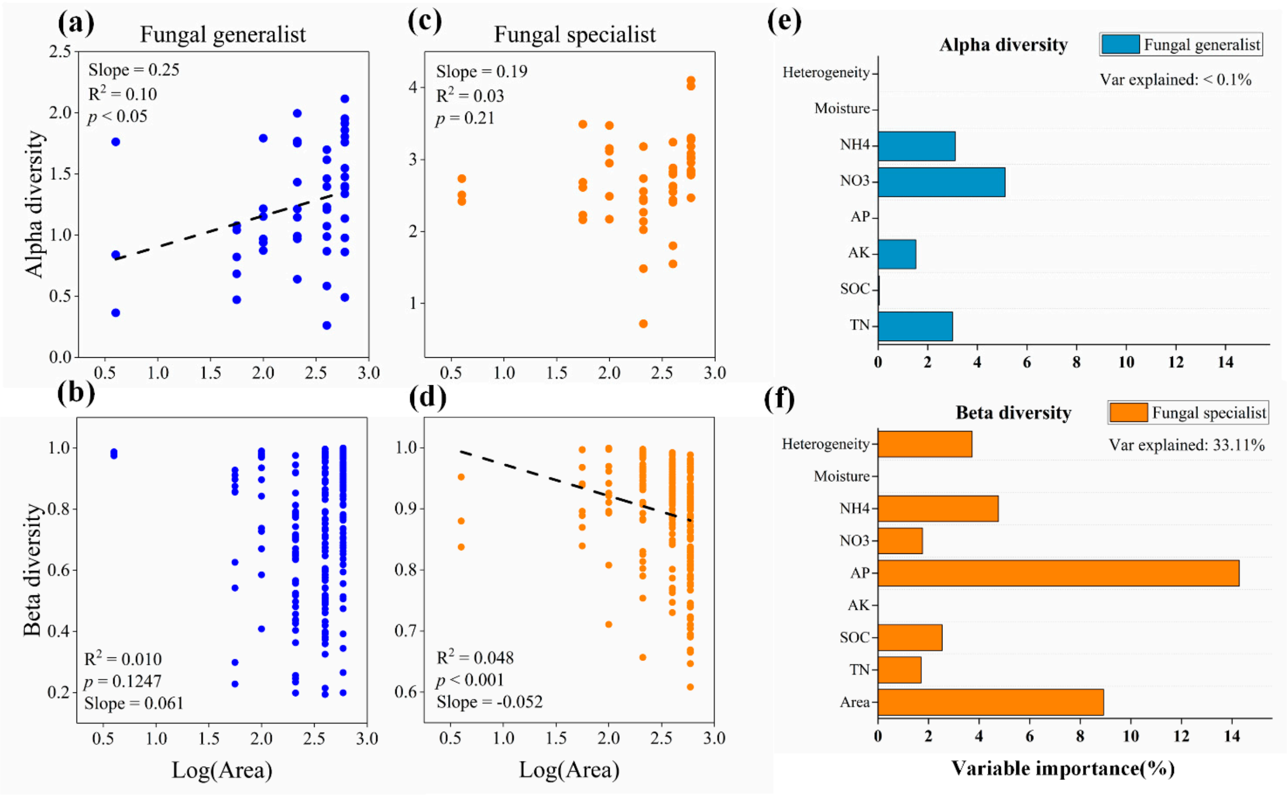

Figure S2 The effects of patch area on the alpha and beta diversity of fungal generalist and specialist, and their important predictors. Panels a and b are for generalist alpha and beta diversity, respectively; panels c and d are for specialist alpha and beta diversity, respectively. Alpha diversity was measured as the Shannon-Weiner index. Beta diversity was measured as average pairwise Bray–Curtis dissimilarities among samples. Panels e and f are for the fraction of the variation in alpha and beta diversity of soil fungal generalist and specialist explained by environmental and spatial predictors. TN: total nitrogen; TP: total phosphorus; AP: available phosphorus; SOC: soil organic carbon; AK: available potassium; NO_3^-: nitrate nitrogen; NH_4^+: ammonium nitrogen; Heterogeneity: the habitat heterogeneity, measured as measured as Euclidean distances of these soil properties among samples.

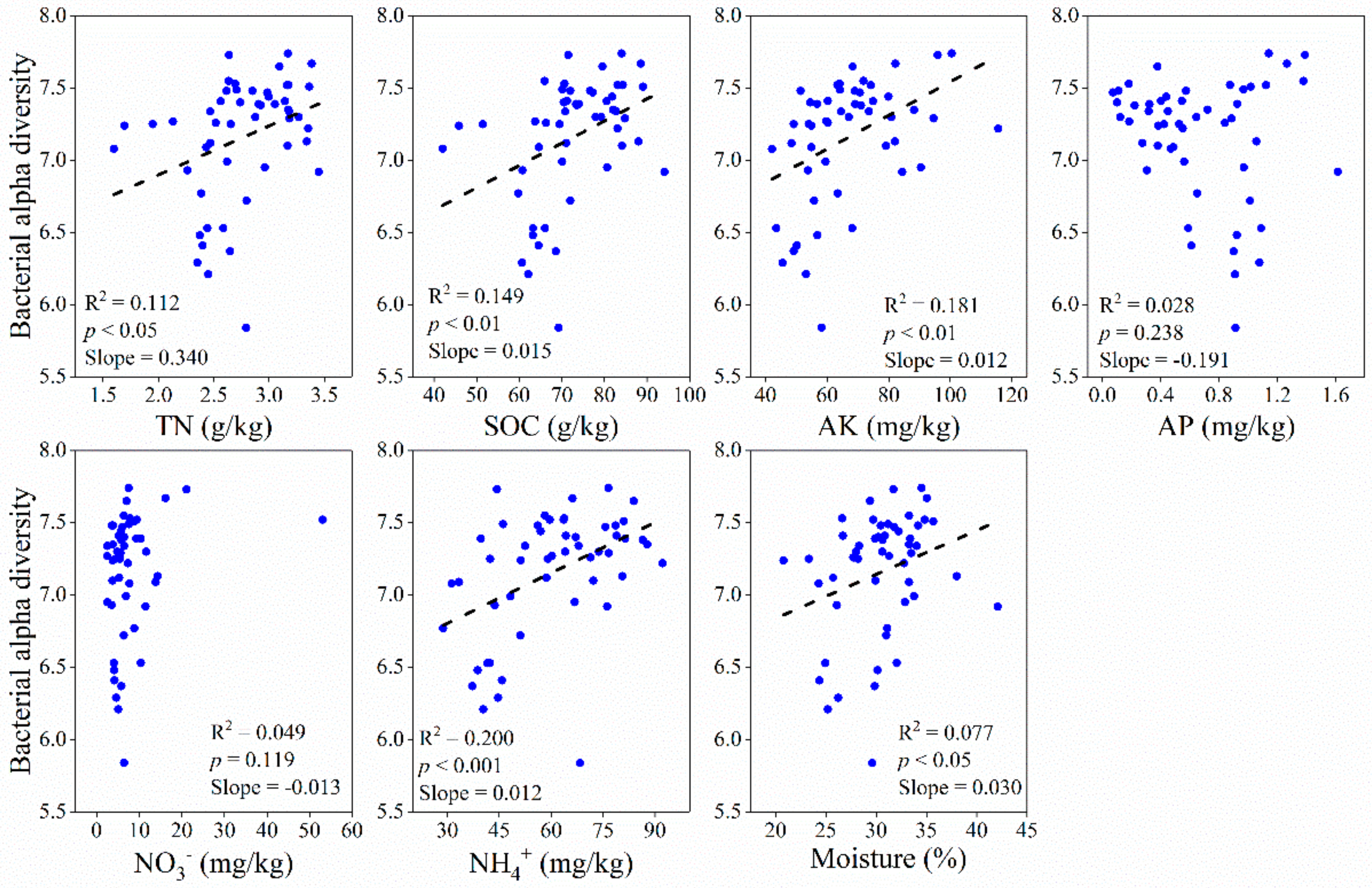

Figure S3 The relationship between soil properties and bacterial alpha diversity.

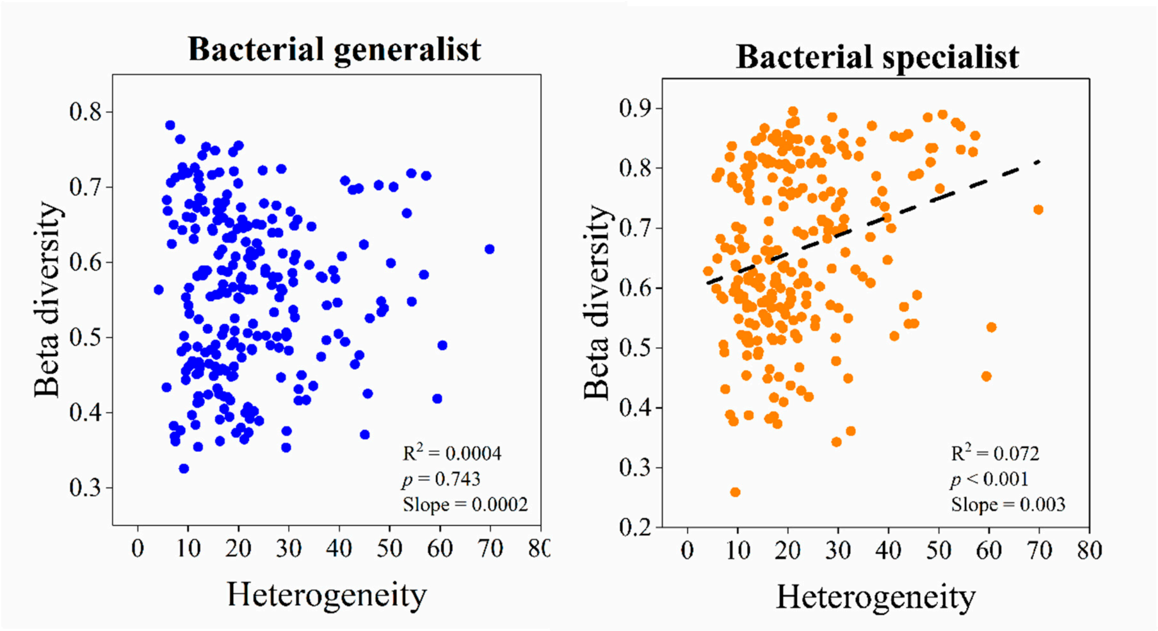

Figure S4 The relationship between soil habitat heterogeneity and beta diversity of bacterial generalist and specialist. The habitat heterogeneity was measured as Euclidean distances of these soil properties among samples.

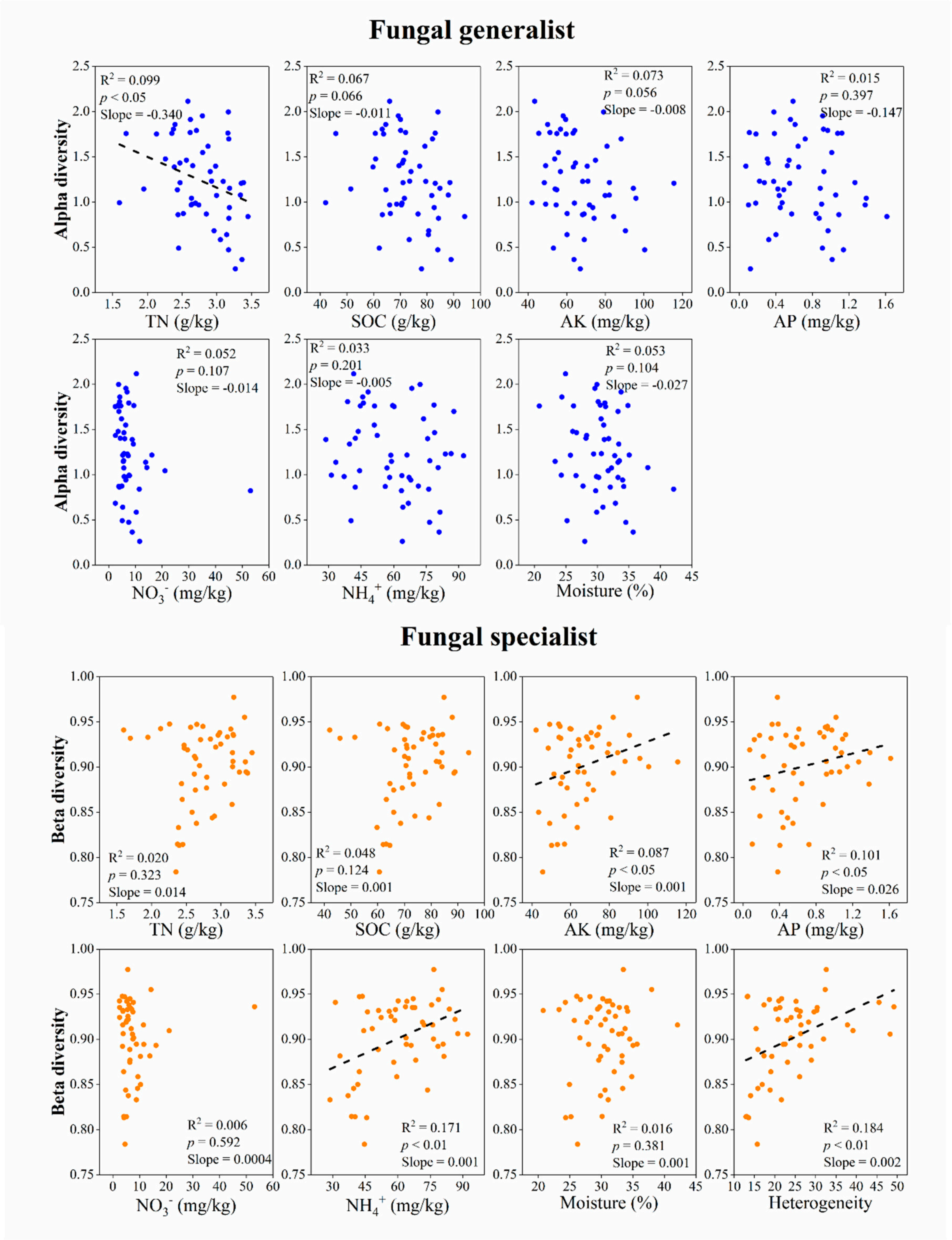

Figure S5 The relationship between soil properties and the diversity of fungal generalist and specialist.

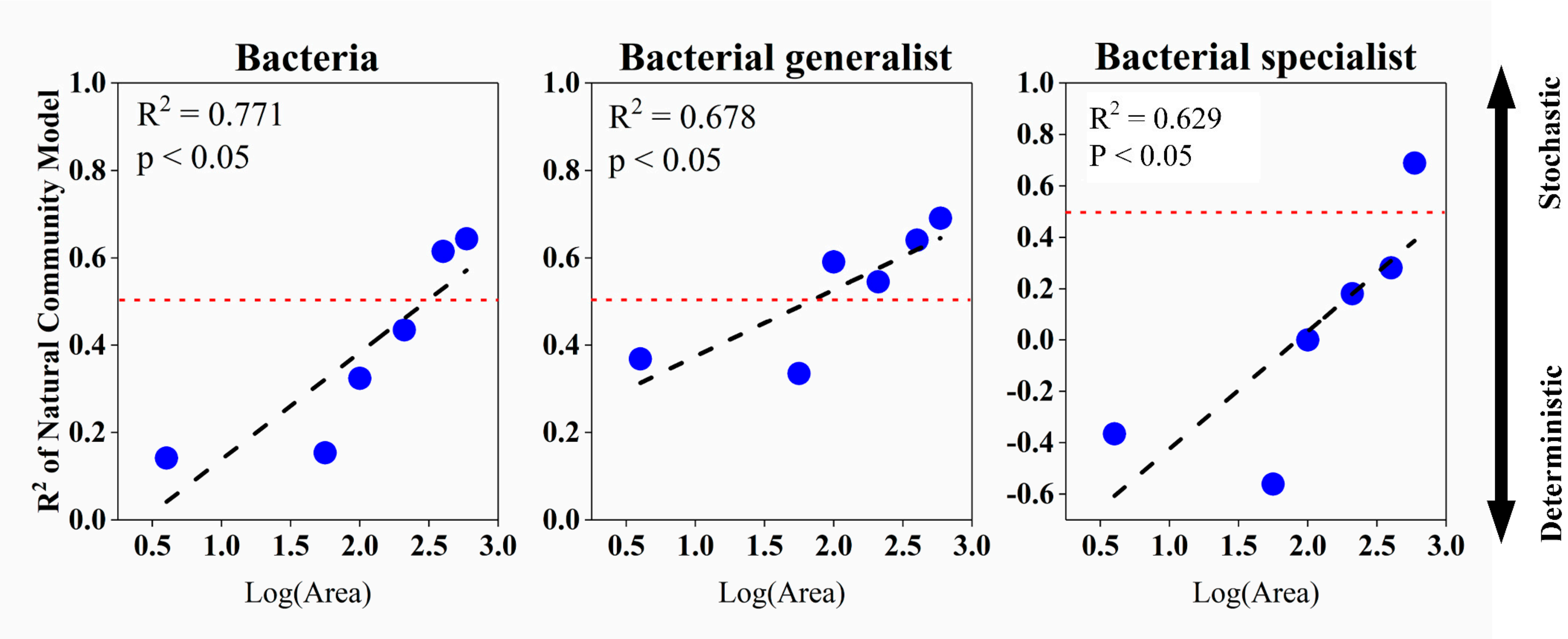

Figure S6 The relationship between patch area and the R^2 value of natural community model of bacterial communities.

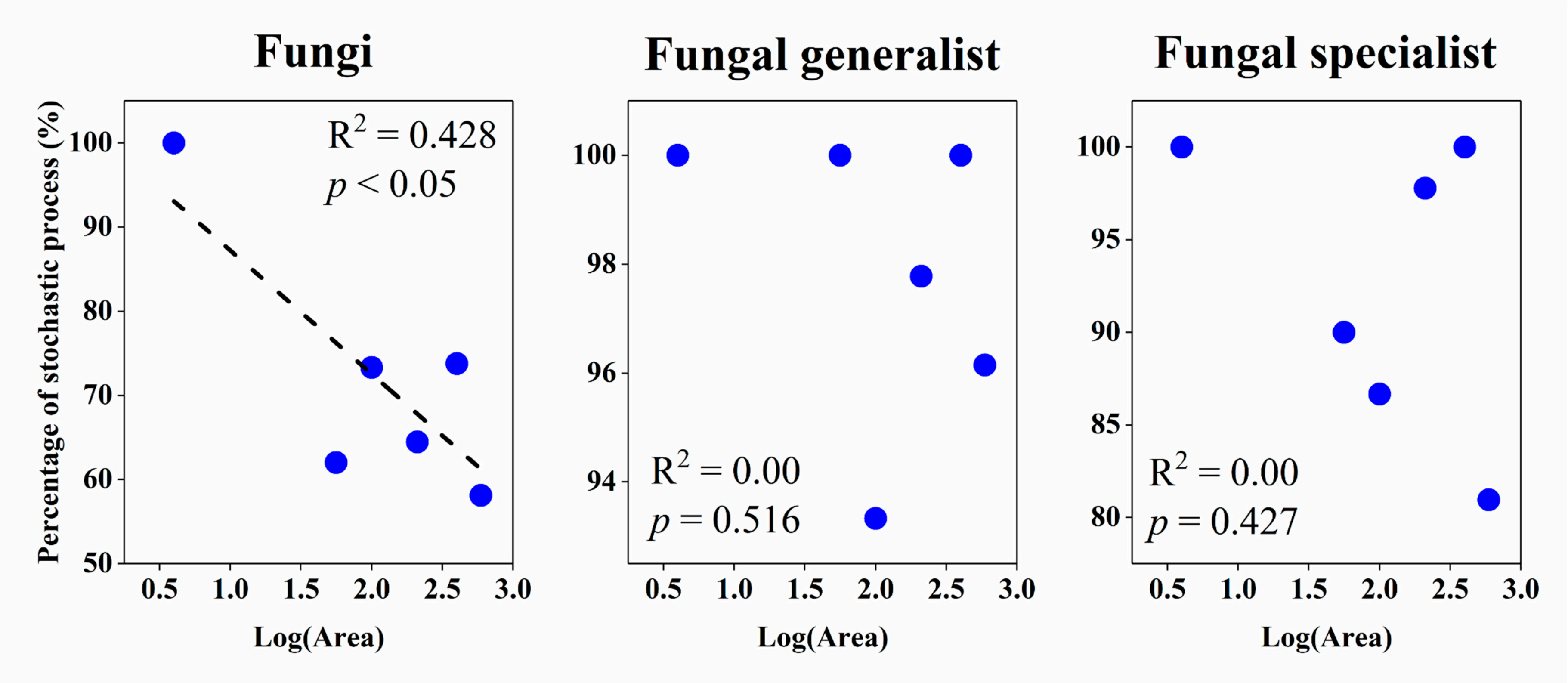

Figure S7 The relationship between patch area and the percentage of stochastic processes in fungal communities.

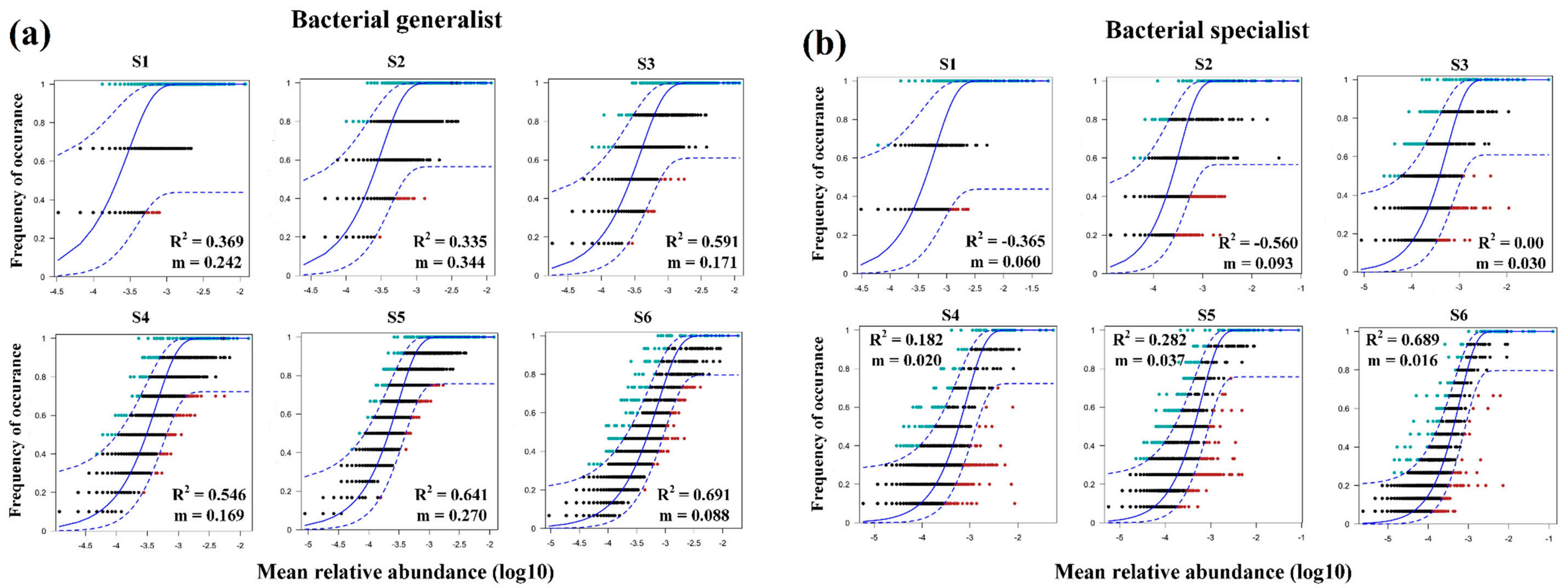

Figure S8 The fit of the neutral community model (NCM) of community assembly. Panel a is for bacterial generalist; panel b is for bacterial specialist. S1-S6 represents patches ranging in size from small to large.

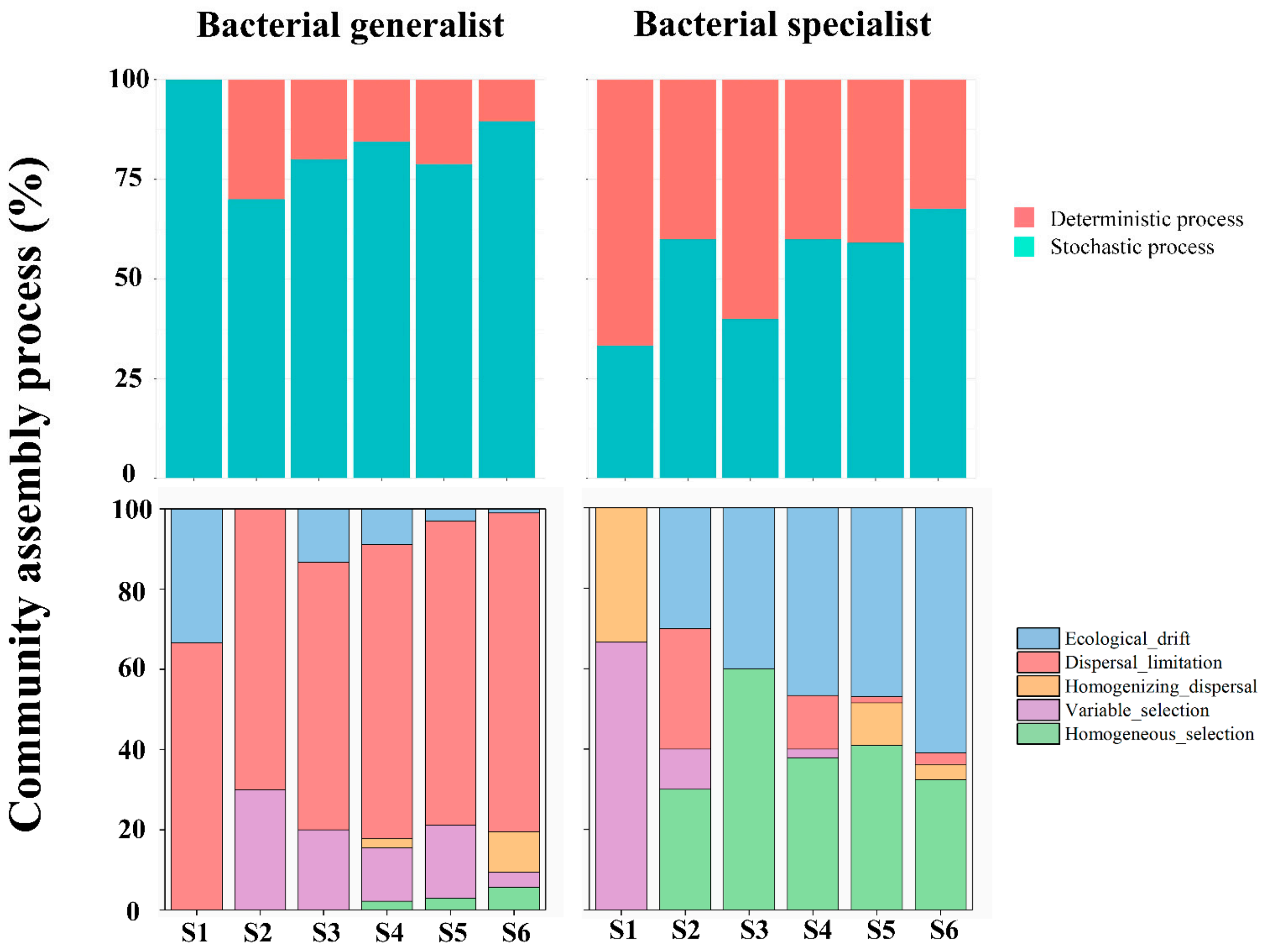

Figure S9 The relative contributions of deterministic, stochastic processes and different ecological processes driving bacterial community assembly. S1-S6 represents patches ranging in size from small to large.

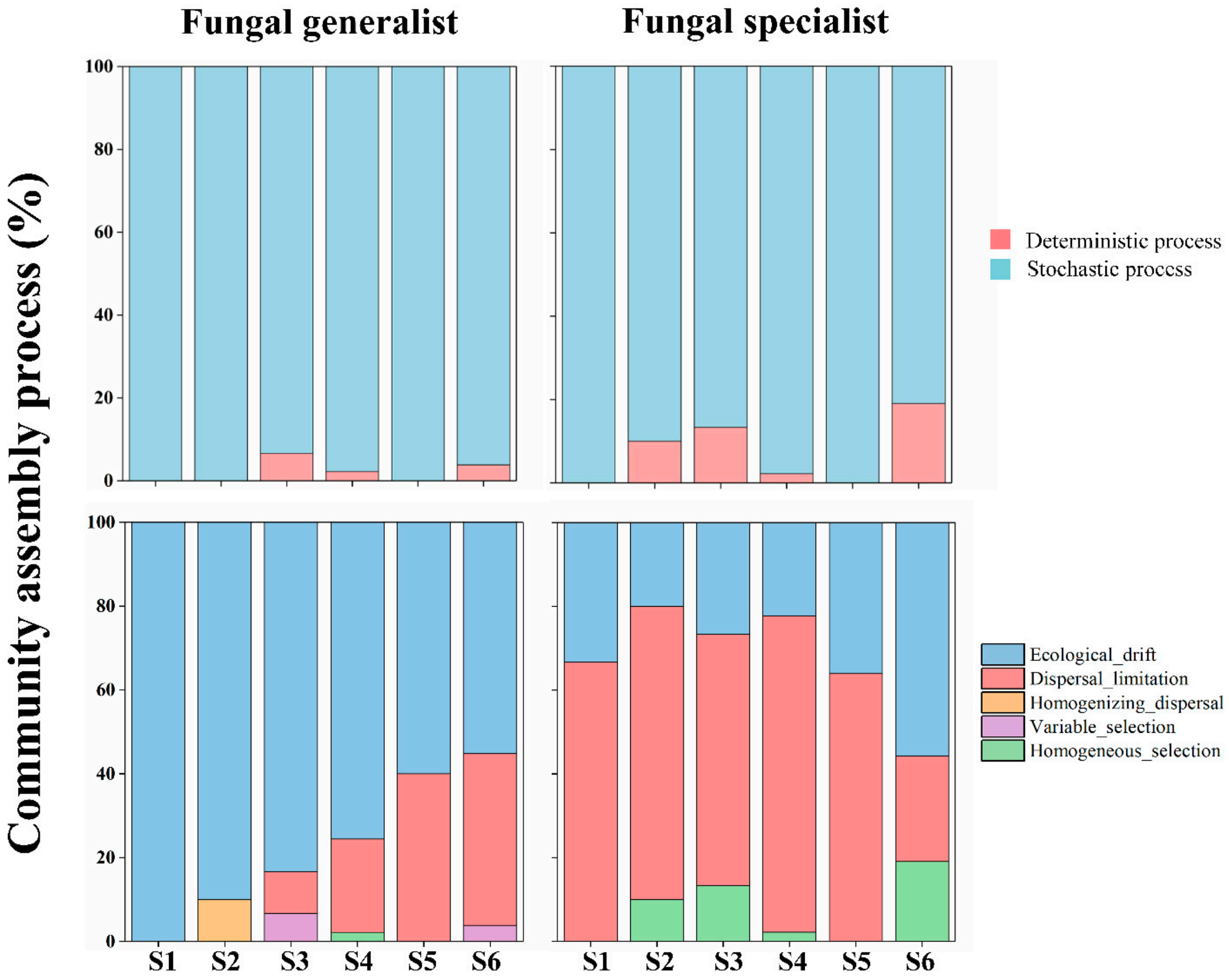

Figure S10 The relative contributions of deterministic, stochastic processes and different ecological processes driving fungal community assembly. S1-S6 represents patches ranging in size from small to large.

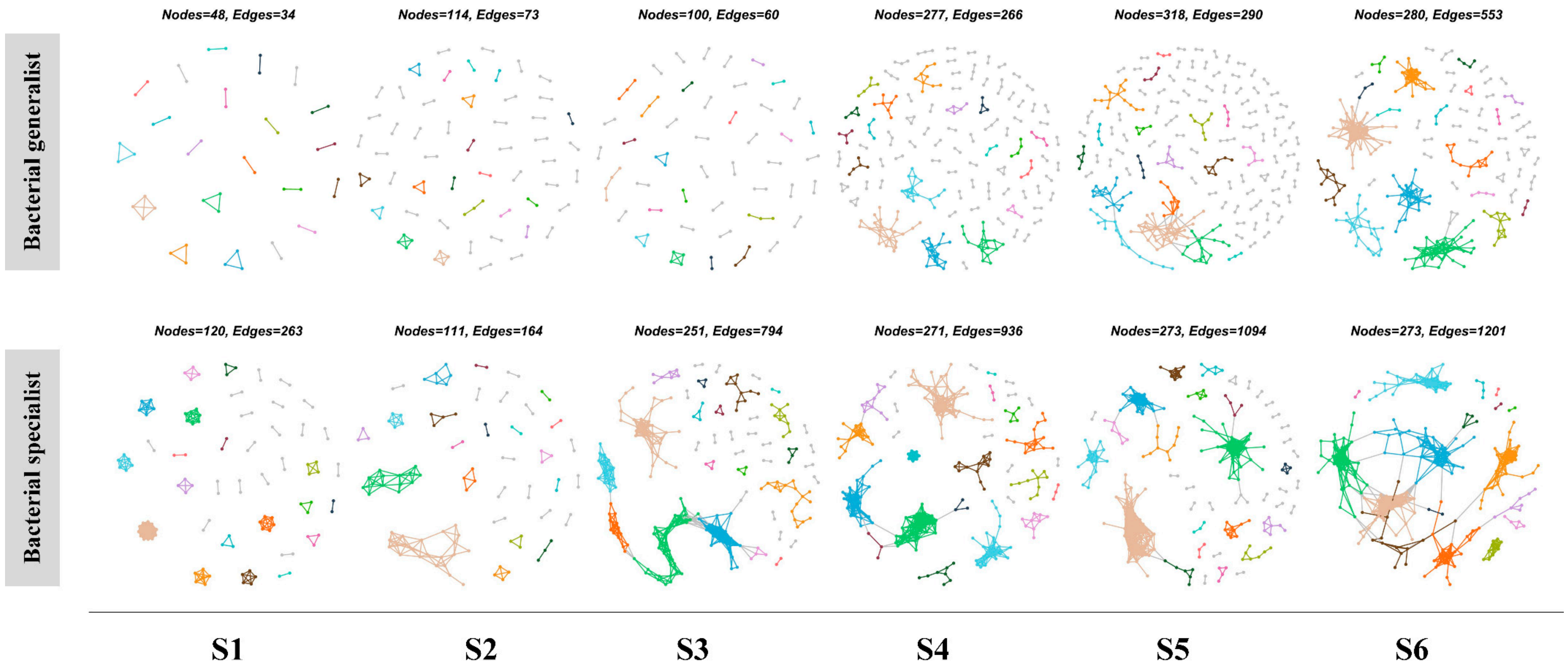

Figure S11 Co-occurrence patterns in soil bacterial generalist and specialist communities on different patches. The top 18 modules in size are shown in different colors, and smaller modules are shown in grey. S1-S6 represents patches ranging in size from small to large.

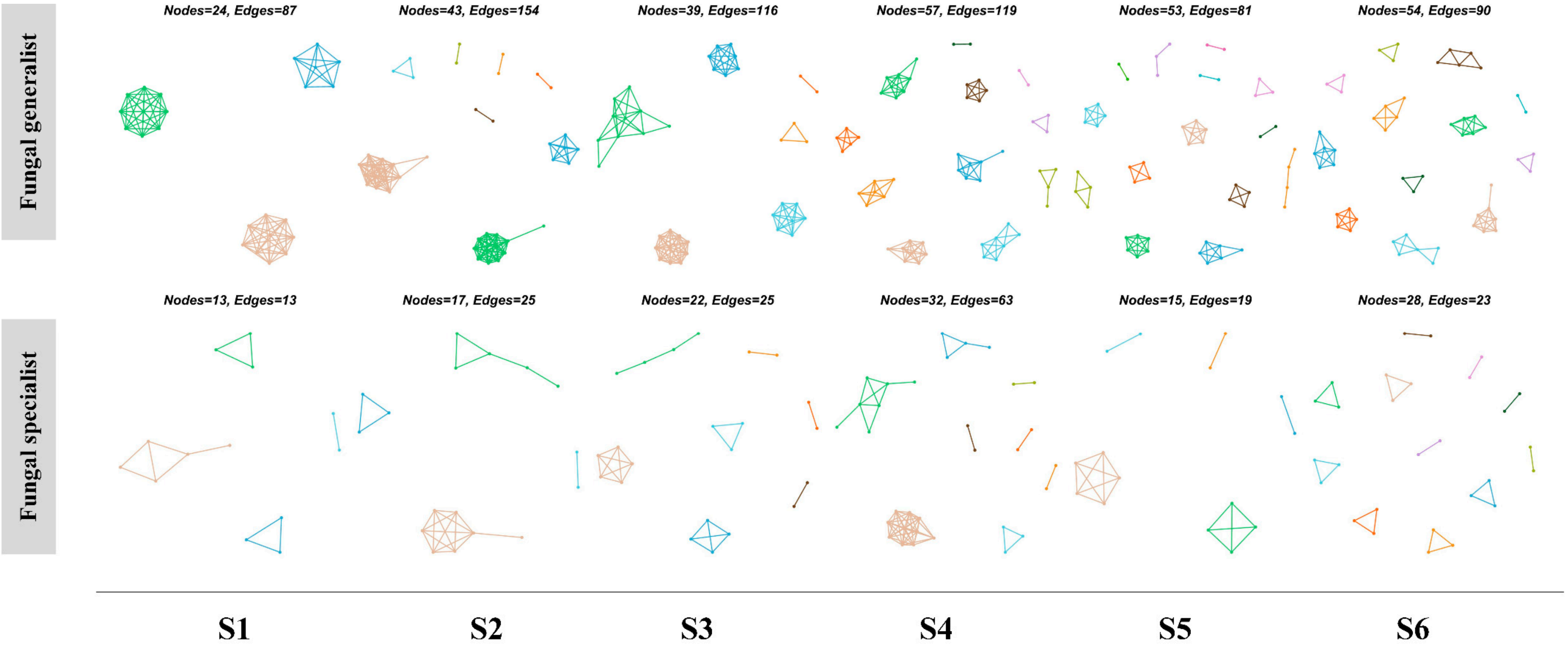

Figure S12 Co-occurrence patterns in soil fungal generalist and specialist communities on different patches. The top 18 modules in size are shown in different colors, and smaller modules are shown in grey. S1-S6 represents patches ranging in size from small to large.

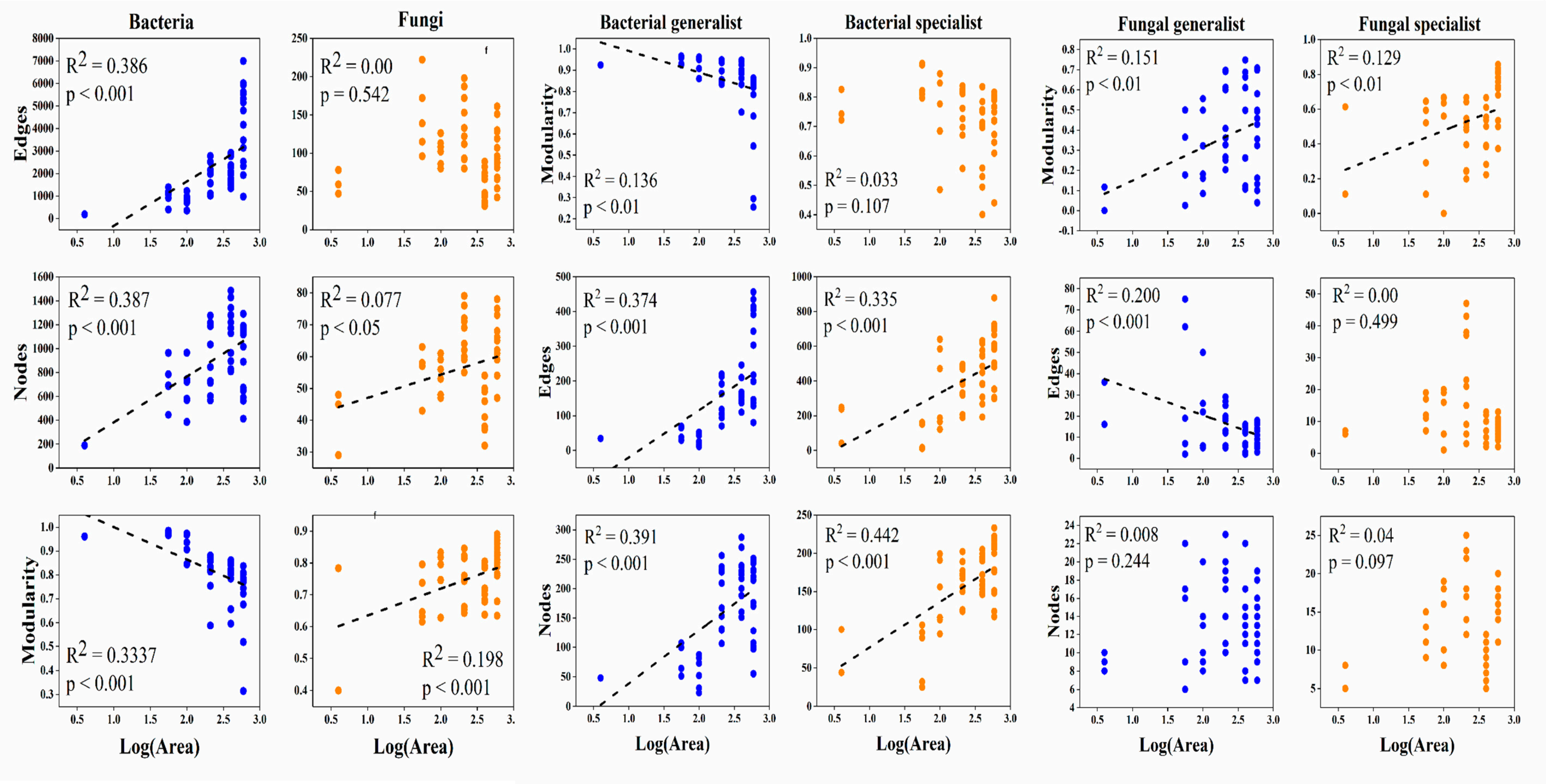

Figure S13 The relationship between patch area and soil bacterial and fungal network properties.
